# The Influence of Reactive Oxygen Species in the Development of Contrast-Induced Nephropathy After Coronary Angiography

**DOI:** 10.3390/jcm14134649

**Published:** 2025-07-01

**Authors:** Hamad Dheir, Gamze Guney Eskiler, Aysel Tocoğlu, Rumeysa Kurt, Emel Gonullu, Ahmet Nalbant, Huseyin Gunduz, Ali Tamer

**Affiliations:** 1 Department of Nephrology, Sakarya University Faculty of Medicine, 54290 Adapazar, Sakarya, Turkey; hamaddheir@sakarya.edu.tr; 2 Department of Medical Biology, Sakarya University Faculty of Medicine, 54290 Adapazar, Sakarya, Turkey; gamzeguney@sakarya.edu.tr; 3 Department of Internal Medicine, Sakarya University Faculty of Medicine, 54290 Adapazar, Sakarya, Turkey; drrumeysak@gmail.com (R.K.); drnalbant@hotmail.com (A.N.); atamer2002@yahoo.com (A.T.); 4 Department of Rheumatology, Sakarya University Faculty of Medicine, 54290 Adapazar, Sakarya, Turkey; emelgonullu@sakarya.edu.tr; 5 Department of Cardiology, Sakarya University Faculty of Medicine, 54290 Adapazar, Sakarya, Turkey; drhuseyingunduz@yahoo.com

**Keywords:** contrast-induced nephropathy, contrast media, inflammation, kidney injury, reactive oxygen species

## Abstract

**Background/Objectives**: Contrast agents can damage renal tissue through multiple mechanisms, particularly by increasing reactive oxygen species (ROS), which contribute to DNA oxidation, lipid peroxidation, and endothelial injury. This prospective, comparative study aimed to evaluate the changes in ROS-related gene expressions—*NFKB1*, *SIRT1*, *NFE2L2*, and *FOXO1*—in patients who developed contrast-induced nephropathy (CIN) following coronary angiography versus those who did not. **Methods**: A total of 48 patients undergoing primary percutaneous coronary intervention were enrolled. Twenty-three patients who developed CIN (Group 1) were compared to 25 matched controls without CIN (Group 2) based on age, gender, and comorbidities. Blood and serum samples were collected 72 h post-contrast exposure to assess biochemical markers and mRNA expression levels of the target genes. **Results**: The mean age was similar between the groups (63 ± 7 vs. 62 ± 6 years; *p* > 0.05), as was gender distribution. Group 1 showed significant increases in serum creatinine and reductions in e-GFR post-procedure. Importantly, *NFKB1*, *NFE2L2*, and *FOXO1* mRNA expression levels were significantly upregulated in CIN patients—by 5.7-, 5.8-, and 4.97-fold, respectively, while *SIRT1* expression was downregulated by 0.76-fold (*p* < 0.05). **Conclusions**: These findings indicate enhanced activation of inflammatory and oxidative stress pathways in CIN patients, particularly through the NF-κB signaling axis. Conversely, reduced *SIRT1* expression suggests diminished antioxidant protection. The study highlights that ROS-related gene expression changes may serve as potential biomarkers for CIN progression. Further studies at the protein level are needed to clarify cytokine roles in these pathways.

## 1. Introduction

Radio-opaque substances are imaging materials that trap X-rays sent into the body, do not allow X-rays to pass through that area, and make hollow organs and vessels more clearly visible [1]. The vast majority of these cells are excreted through the kidneys and can damage kidney tissue temporarily or permanently through many mechanisms [2,3]. In particular, exposure to radio-opaque substances causes the release of vasoconstrictor mediators, systemic hypovolemia, hyperviscosity, and increased osmotic load in the distal tubule, decreased renal blood flow, decreased oxygen delivery to renal tissue, and increased oxygen consumption [4,5]. This may result in direct cytotoxicity and hypoxia in the renal medulla, leading to renal dysfunction. Furthermore, renal dysfunction occurs due to endogenous biochemical damage, such as released free oxygen radicals and decreased antioxidant enzyme activity.

Cellular reactive oxygen species (ROS) are tightly controlled by different mechanisms to maintain normal cellular function [5]. Increased ROS due to oxidative damage causes DNA oxidation, lipid peroxidation, and endothelial cell damage, and this process is considered the primary mechanism of damage in most tissues [5]. The transcription factor nuclear factor kappa B (NF-κB), which plays a vital role in cellular viability and inflammation, can be activated by H_2_O_2_, which is known to be sensitive to cellular oxidative status and contribute to cell death. SIRT1 plays a role in the ROS mechanism by regulating its key regulator, NF-κB [4]. *SIRT1* is a gene in the cell nucleus that contributes to cellular regulation. *SIRT1* inhibits NF-κB-regulated gene expression by deacetylating the RelA/p65 subunit of NF-κB [6]. *SIRT1* is also associated with various molecular mechanisms, including the inhibition of p53, regulation of the Forkhead box gene (FOXO), and Nuclear factor erythroid-2-related factor 2 (NRF2) activity coded by the *NFE2L2* gene [7]. FOXO plays a role in the ROS mechanism by upregulating catalase and SOD enzymes involved in the detoxification of ROS [4,5]. NRF2 is a crucial transcription factor that can stimulate the expression of antioxidant and detoxification enzymes and provide a cellular response to oxidative stress. Under normal conditions, NRF2 is retained in the cytoplasm by a group of rapidly degrading proteins. Under oxidative stress, NRF2 transfers to the nucleus, binds to the DNA promoter, and initiates the transcription of antioxidative genes and proteins. Heme oxygenase-1 (HO-1) is one of the genes whose transcription is initiated by NRF2. HO-1 is a human gene encoding the enzyme heme oxygenase 1. It reduces heme to bilirubin, CO, and iron, which have antioxidant and anti-inflammatory properties [7,8].

Contrast-induced nephropathy (CIN) is detected following intravascular administration of contrast media (CM). The pathophysiological molecular mechanisms of CIN could be associated with direct or indirect effect, and generation of ROS [4,5]. In this context, there could be a relationship between inflammation and the progression of CIN due to CM inducing inflammation.

In this context, the present study aimed to comparatively determine the changes in ROS-related *NFKB1*, *SIRT1*, *NFE2L2*, and *FOXO1* expression levels in patients who developed CIN after primary percutaneous coronary intervention (p-PCI) and in patients who did not develop CIN.

## 2. Materials and Methods

### 2.1. Selection of the Study Group and Collection of Blood Samples

The prospective study was conducted on a total of 48 patients underwent p-PCI. The study included 23 patients with CIN (Group 1) and 25 patients without CIN (Group 2). This study was conducted over a 3-month period between March 2021 and June 2021, involving patients who underwent p-PCI. Among the 213 patients who agreed to participate, 97 were excluded due to failure to complete the 28-day follow-up. Among the remaining 116 patients who successfully completed follow-up, CIN was detected in 23 patients. From the 91 patients who did not develop CIN, 25 were randomly selected and matched with the CIN group in terms of age, sex, and comorbidities to serve as the control group. Patients younger than 75 years, without a diagnosis of chronic kidney disease (CKD), and who signed the informed consent form were included in the study. Patients were excluded if they were 75 years or older, had a diagnosis of CKD, had experienced an infection and/or inflammatory disease within the last 3 months, had been exposed to CM within the last 5 days, had a history of acute coronary syndrome, had active malignancy, had a hemoglobin level <7 mg/dL, or developed acute kidney injury during follow-up due to causes other than CIN. Both groups were matched for age, gender, and comorbid conditions. Blood and serum samples were collected 3 days after the contrast agent administration and stored at −80 °C. Blood samples were collected at baseline (pre-procedural), and on the 72nd hour, 7th day, 14th day, and 28th day after the procedure. Demographic characteristics and biochemical parameters of the patients were recorded. Written informed consent was obtained from all participating patients, and ethics committee approval of our university was obtained (no = E-16214662-050.01.04-1651448). CIN was defined as an increase in serum creatinine of at least 25% from baseline and/or an absolute increase of more than 0.5 mg/dL. Iohexol (Omnipaque^®^) was used as the CM during coronary intervention. The amount of contrast administered varied based on the clinical judgment of the interventional cardiologist and the scope of the procedure.

### 2.2. Reverse Transcriptase-Polymerase Chain Reaction (RT-PCR) Analysis

Changes in the expression levels of *NFKB1*, *SIRT1*, *NFE2L2*, and *FOXO1* were analyzed by RT-PCR analysis. In this context, RNA isolation was performed from Group 1 and 2 patients of blood at 28th day by TRIzol (Thermo Scientific, Waltham, MA, USA). The concentration of the isolated RNA was determined by the Qubit system (Invitrogen, Waltham, MA, USA). cDNA was synthesized from the obtained RNA via a high-capacity cDNA kit (Thermo Fisher Scientific, USA). The mRNA levels of *NFKB1*, *SIRT1*, *NFE2L2*, and *FOXO1* were analyzed by RT-PCR (Reverse Transcription Polymerase Chain Reaction-Applied Biosystems, USA) by using TaqMan probes (Thermo Fisher Scientific, Waltham, MA, USA), and beta-Actin was used as an internal control. Changes in the expression levels of the genes were defined via data analysis programs (REST (2009 V2.0.13)).

### 2.3. Statistical Analysis

The statistical analysis of the dataset was conducted using IBM SPSS version 21. Both the Kolmogorov–Smirnov and Shapiro–Wilk tests were employed to assess the normality of numerical data. Variables with a normal distribution were presented as mean ± standard deviation and compared using the student’s *t*-test. Variables that did not follow a normal distribution were expressed as median (25th–75th percentiles) and compared using the Mann-Whitney U test. Categorical variables were summarized as frequencies (percentages), and comparisons between groups were performed using chi-square tests. In the comparison of two dependent continuous numerical variables, the paired sample t-test was used when the data followed a normal distribution, whereas the Wilcoxon signed-rank test was applied in cases where the data did not exhibit normality. Receiver Operating Characteristic (ROC) curve analysis was used to evaluate the prognostic values of *NFKB1*, *SIRT1*, *NFE2L2*, and *FOXO1*. The cut-off values were determined based on the highest sensitivity and specificity. A *p*-value of <0.05 was considered statistically significant in all analyses.

## 3. Results

### 3.1. Clinical Features of Patients

A total of 48 patients, 23 with CIN (Group 1) and 25 without CIN (Group 2), were included in the study. The mean age of the patients was 63 ± 7 years in Group 1 and 62 ± 6 years in Group 2 (*p* > 0.05), respectively, and in terms of male gender, there were 16 (69.6%) males in Group 1 and 17 (68.0%) in Group 2 (*p* > 0.05). There was no significant difference in the comorbidity of diabetes mellitus, hypertension, heart failure, or more than one comorbid condition (Table 1). However, CIN developed more in patients with >3 comorbid conditions (*p* = 0.03). At baseline, serum uric acid was higher in Group-1 (6.9 ± 1.8 mg/dL vs. 5.1 ± 1.3 mg/dL, *p* = 0.004), and serum albumin level was significantly lower in the CIN group compared to the control group (3.6 ± 0.4 vs. 3.9 ± 0.3 g/L, *p* = 0.005). The mean serum creatinine of the patients who developed CIN was 2.06 (1.52–3.07) mg/dL and e-GFR: 31.16 ± 16.09 mL/min/1.73 m^2^ (*p* < 0.001) (Table 2). Additionally, the comparison of the baseline values of CIN patients with 72 h after CM exposure were summarized in Table 3. There was significant difference in the serum urea, creatinine, eGFR, calcium and albumin levels between baseline and 72 h exposure.

### 3.2. Evaluation of Changes in the Inflammation-Related Gene Levels

**Figure 1:**
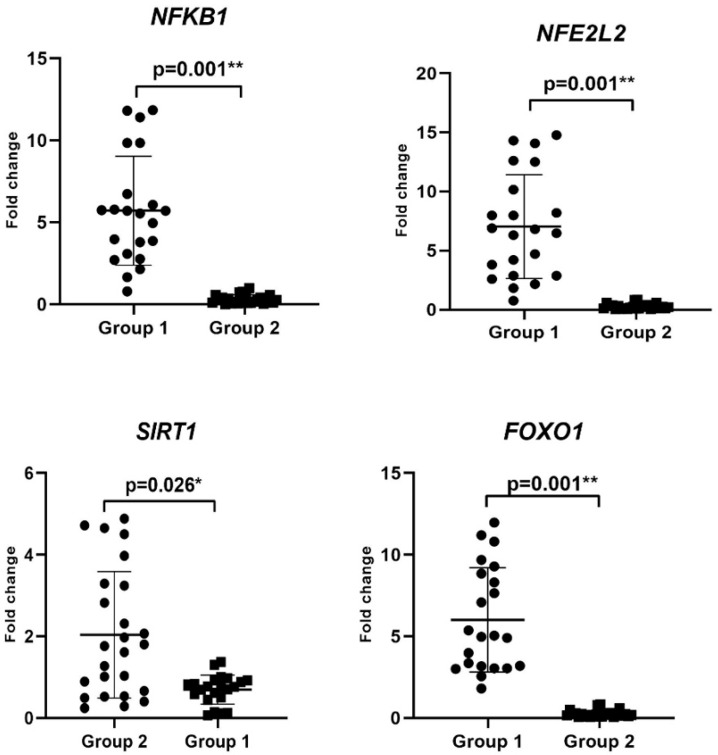
Comparison of expression of *NFKB1*, *NFE2L2*, *FOXO1*, and *SIRT1* levels by RT-PCR analysis in Group 1 and Group 2 patients (*p* < 0.05 *, *p* < 0.01 **).

The changes in the expression levels of *NFKB1*, *SIRT1*, *NFE2L2*, and *FOXO1*, associated with ROS involved in the mechanism of development of CIN, were determined by RT-PCR analysis (Figure 1, Table 4). *NFKB1*, *NFE2L2*, and *FOXO1* mRNA levels increased 5.7-fold (3.08–8.54), 5.8-fold (2.88–10.16), and 4.97-fold (3.04–8.84), respectively, in Group 1 (*p* = 0.001), while *SIRT1* expression level decreased by 0.76-fold (0.5–0.92) (*p* = 0.002) compared with the Group 2 (Table 4). Therefore, the upregulation of *NFKB1*, *NFE2L2*, and *FOXO1* mRNA levels could predict CIN progression and induce inflammation.

### 3.3. Association of ROS-Related Gene Levels with CIN in the Patients

**Figure 2:**
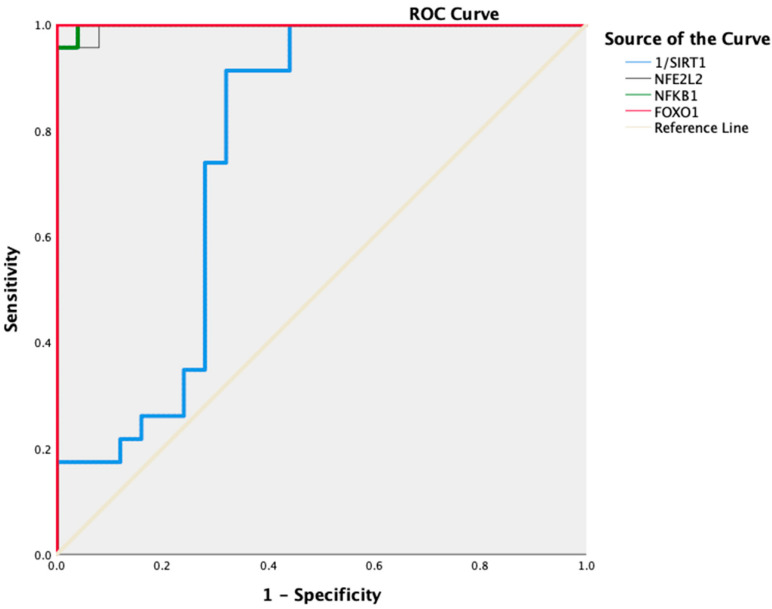
ROC curve histogram of *NFKB1*, *NFE2L2*, *FOXO1*, and *SIRT1* expression levels in Group 1 and Group 2 patients.

We performed ROC curve analysis to assess the prediction of changes in the mRNA levels of *NFKB1*, *SIRT1*, *NFE2L2*, and *FOXO1* for CIN progression. According to ROC analysis, with respect to increasing the risk of developing CIN, we found that 96% sensitivity and 100% specificity for *NFE2L2* with 1.34- (AUC: 0.977; 95% CI: 0.987–1; *p* < 0.001), 100% sensitivity and 96% specificity for *NFKB1* with 0.78-fold (AUC: 0.998; 95% CI: 0.993–1; *p* < 0.001), 100% sensitivity and 100% specificity for *FOXO1* with 1.32-fold (AUC: 1.000; 95% CI: 1–1; *p* < 0.001). Additionally, the risk of CIN increased at values below 0.88-fold (AUC: 0.763; 95% CI: 0.620–0.907; *p* = 0.002) with 74% sensitivity and 72% specificity for *SIRT1* (Figure 2).

Furthermore, the cut-off expression levels of *NFE2L2* > 1.34-fold, *NFKB1* > 0.78-fold and *SIRT1* < 0.88-fold predicted an increased risk of CIN by 26.32 (95% Cl: 3.731–166.667; *p* < 0.001), 11.5 (95% Cl: 3.043–43.461; *p* < 0.001) and 2.36 (95% Cl: 1.216–4.587; *p* = 0.004) times, respectively. In addition, the risk of CIN was fully predicted for *FOXO1* with the cut-off value > 1.32, and the odds ratio was calculated as infinite (OR = ∞) (*p* < 0.001) (Table 5).

## 4. Discussion

This prospective, matched, and comparable study reported the changes in the expression levels of *NFKB1*, *SIRT1*, *NFE2L2*, and *FOXO1* associated with ROS in patients who underwent p-PCI and developed CIN. To our knowledge, for the first time, we have shown the impact of inflammation-related biomarkers at the gene level in cardiac patients who develop CIN. Our findings suggest that *NFKB1*, *NFE2L2*, and *FOXO1* expression levels were significantly upregulated due to increased ROS and could predict the risk of CIN progression. Our findings, in agreement with the in vitro and in vivo studies [9,10], supported the view that NF-κB activates inflammatory pathways and may trigger CIN development by increasing ROS production.

Patients with CIN have a marked decrease in e-GFR and develop a non-oliguric reversible acute kidney injury without the need for dialysis. These findings suggest that the development of CIN is associated with significant renal dysfunction. Additionally, CIN is frequently associated with high creatinine levels [11]. In our study, Group 1 patients had high creatinine levels with decreased e-GFR. Our results were consistent with the literature.

Our study population consisted of 75% of patients with type II diabetes mellitus. Increased NF-κB activation in type II diabetes mellitus has also been implicated in impaired vascular function, including myogenic tone, vascular reactivity, and inflammatory response through the detection of PARP-1, SP-1, and COX-2 activity [12]. According to studies on Resolvins, naturally occurring polyunsaturated fatty acids have anti-inflammatory effects in various tissues, including the kidneys. Resolvins exert this anti-inflammatory effect in diabetic patients by suppressing inflammatory responses by (1) inhibition of nucleotide-binding oligomerization domain protein 3 inflammatory, (2) inhibition of NF-κB molecular pathways, (3) amelioration of oxidative stress, (4) modulation of nitric oxide synthesis/release, and (5) prevention of local and systemic leukocytosis [12,13]. In this context, NF-κB plays a crucial role in the pathogenesis of diabetic nephropathy and metabolic kidney diseases. Furthermore, patients with glomerular damage exhibit a higher level of NF-κB protein. NF-κB overexpression is detected in IgA nephropathy, particularly in patients with high proteinuria and decreased renal function [14,15]. In the present study, we assessed that higher expression levels of *NFKB1* were detected in Group 1 compared with Group 2 and predicted the risk of CIN progression.

NRF2 is a nuclear transcription factor that stimulates the expression of genes involved in the transcription and replication of mitochondrial DNA and the oxidative phosphorylation system. Furthermore, NRF2 regulates the expression of various genes responsible for cellular detoxification, antioxidant function, anti-inflammation, drug/xenobiotic transport, and stress-related factors, as well as a protective role against acute kidney injury (AKI) [16]. In the literature, the role of NRF2 has been demonstrated with different AKI models. In the kidneys, NRF2 plays a dynamic role in ameliorating renal damage caused by ROS production [17]. Liu et al. showed that renal function, histology, vascular permeability, and survival in NRF2-/- mice under ischemic conditions blocked by NAC or glutathione treatment were significantly reduced compared to the control group [18]. To our knowledge, we, for the first time, demonstrated the association between NRF2 and developing CIN in patients. Our findings showed that the upregulation of *NFE2L2* expression level predicted the risk of CIN with 96% sensitivity and 100% specificity, and could be a biomarker for the diagnosis of early stages of CIN.

Similarly, in our study, changes in the expression level of *FOXO1* (1.32-fold with 100% specificity (AUC: 1.000; 95% CI: 1–1; *p* < 0.001) could be a potential diagnostic biomarker for CIN development. FOXO has many vital functions, e.g., it is an important downstream factor of the PI3K/Akt signaling pathway that induces the expression of death receptor ligands and Bcl-2 family members through negative regulation of the PI3K-Akt signaling pathway and controls cell survival, proliferation, and angiogenesis [18,19,20]. *Abelmoschus manihot* (L.) Medik. (Malvaceae), a plant used in Traditional Chinese Medicine to treat some kidney diseases, has been shown to treat CIN through some pathways, including FOXO [21]. Furthermore, FOXO mediated by SERPINB1 decreases ROS production and MDA levels in a diabetic nephropathy in vitro model [22]. In addition, peroxisome proliferator-activated receptor gamma-assisted activating factor-1α Forkhead-box transcription factor (PGC-1α FOXO) causes oxidative stress and apoptosis [23]. In the current study, we, for the first time, analyzed the upregulation of *FOXO1* mRNA levels due to higher ROS production in Group 1 patients. Thus, the findings obtained in the present study suggest that increased *FOXO1* expression levels in CIN patients may lead to damage to renal function. However, further molecular investigations are required to assess the role of FOXO-associated signaling pathway in CIN progression.

Furthermore, a decreased *SIRT1* mRNA level was detected in Group 1 patients. SIRT1 is known as a deacetylase that plays a protective role under cellular stress conditions. The activation of SIRT-1 ameliorates some metabolic diseases associated with many other molecular pathways. In addition, SIRT1 has also been shown to attenuate diabetic nephropathy in vitro and in vivo experimental models of diabetes involving podocytes, mesangial cells, and renal proximal tubular cells [24,25]. In addition, the nephroprotection of SIRT1 in diabetic nephropathy is mediated by deacetylation of some transcription factors, including FoxO, RelA/NF-kβ, STAT-3, and PGC-1α/PPARγb, etc., and the higher activity of SIRT1 exerts the renal protective effect for diabetic kidney disease [25]. In this context, our results suggest that the downregulation of *SIRT1* could be associated with the impairment of nephprotective function, uncontrolled oxidative stress, and increased cellular damage.

## 5. Conclusions

In conclusion, our study provides important contributions to our understanding of the role of ROS-mediated inflammation in CIN development and the molecular basis of these mechanisms. Our findings showed that the higher activation of the NF-κb signaling pathway and the upregulation of *NFE2L2* and *FOXO1* levels were observed in CIN patients with the downregulation of *SIRT1*. These findings suggest that CIN development leads to inflammation and ROS production, and changes in the expression levels of *NFKB1*, *SIRT1*, and *NFE2L2* could predict the risk of CIN in patients. However, further studies can be carried out in more comprehensive CIN patient groups to verify changes in the gene expression level at the protein level. Additionally, the role of inflammation, ROS-mediated signaling pathways, and cytokine levels should be investigated.

## Figures and Tables

**Table 1:** Comparison of patient groups according to baseline demographic parameters.

	All Patients n = 48	Group 2 n = 25	Group 1 n = 23	*p*-Value
Age, years	63 ± 7	62 ± 6	63 ± 7	0.478
Male sex, n (%)	33 (68.8%)	17 (68%)	16 (69.6%)	0.907
Body Mass Index, kg/m^2^	28.86 ± 5.36	28.87 ± 6.41	28.85 ± 4.07	0.990
**Smoking status, n (%)**				
Yes	22 (45.8%)	11 (44%)	11 (47.8%)	0.756
No	17 (35.4%)	10 (40%)	7 (30.4%)	
**Alcohol use, n (%)**				
Yes	4 (8.30%)	3 (12%)	1 (4.30%)	0.583
No	37 (77.1%)	19 (76%)	18 (78.3%)	
**Comorbidities, n (%)**				
Diabetes mellitus	36 (75%)	16 (64%)	20 (87%)	0.670
Hypertension	20 (41.7%)	8 (32%)	12 (52.2%)	0.157
Coronary artery disease	24 (50%)	15 (60%)	9 (39.1%)	0.149
Heart failure	9 (18.8%)	3 (12%)	6 (26.1%)	0.279
Hyperlipidemia	25 (52.1%)	10 (40%)	15 (62.2%)	0.081
**Number of comorbidities, n (%)**				
0	8 (16.7%)	6 (24%)	2 (8.7%)	0.122
1	9 (18.8%)	6 (24%)	3 (13%)	
2	3 (6.3%)	2 (8%)	1 (4.3%)	
3	13 (27.1%)	8 (32%)	5 (21.7%)	
4	9 (18.8%)	2 (8%)	7 (30.4%)	
5	3 (6.3%)	1 (4%)	2 (8.7%)	
<3	33 (68.8%)	22 (88%)	11(47.8%)	0.03
3≥	15 (31.3%)	3 (12%)	12 (52.2%)	

**Table 2:** Comparison of laboratory parameters of patients at baseline and 72 h after CM exposure.

	All Patients n = 48	Group 2 n = 25	Group 1 n = 23	*p*-Value
**Baseline**				
Serum Urea, mg/dL	35 (27–49)	30 (25–36)	43 (30–77)	0.001
Serum Uric acid, mg/dL	6 ± 1.8	5.1 ± 1.3	6.9 ± 1.8	0.004
Serum Creatinine, mg/dL	0.80 ± 0.17	0.76 ± 0.17	0.84 ± 0.16	0.139
eGFR, mL/min/1.73 m^2^	95.96 ± 15.31	98.17 ± 12.84	93.56 ± 17.60	0.303
Serum Sodium, mEq/L	137 ± 3	138 ± 3	136 ± 4	0.216
Serum Potassium, mmol/L	4.4 ± 0.4	4.3 ± 0.3	4.4 ± 0.5	0.245
Serum Calcium, mg/dL	9.2 ± 0.5	9.2 ± 0.5	9.1 ± 0.6	0.562
Serum Albumin, g/L	3.7 ± 0.4	3.9 ± 0.3	3.6 ± 0.4	0.005
**72nd hour**				
Serum Urea, mg/dL	62 ± 44	32 ± 10	95 ± 44	<0.001
Serum Uric acid, mg/dL	6.7 ± 2.6	5.3 ± 1.3	8.5 ± 2.8	<0.001
Serum Creatinine, mg/dL	1.1 (0.84–2.05)	0.87 (0.71–0.99)	2.06 (1.52–3.07)	<0.001
eGFR, mL/min/1.73 m^2^	63.26 ± 34.48	92.79 ± 13.94	31.16 ± 16.09	<0.001
Serum Sodium, mEq/L	137 ± 4	139 ± 2	135 ± 5	0.002
Serum Potassium, mmol/L	4.3 (4.1–4.8)	4.4 (4.3–4.8)	4.2 (3.8–4.4)	0.017
Serum Calcium, mg/dL	9.2 (8.5–9.6)	9.6 (9.3–9.9)	8.5 (7.9–8.8)	<0.001
Serum Albumin, g/L	3.6 (3.3–4.2)	4.1 (3.9–4.2)	3.2 (3.1–3.6)	<0.001

Abbreviation: eGFR: estimated glomerular filtration rate.

**Table 3:** The comparison of the CIN group’s baseline values with 72 h after CM exposure.

Group 1 n = 23	Baseline	72nd hour	*p*-Value
Serum Urea, mg/dL	43 (30–77)	94 (60–122)	<0.001
Serum Uric acid, mg/dL	6.9 ± 1.8	8.5 ± 2.8	0.162
Serum Creatinine, mg/dL	0.81 (0.76–0.95)	2.06 (1.52–3.07)	<0.001
eGFR, mL/min/1.73 m^2^	93.56 ± 17.60	31.16 ±16.09	<0.001
Serum Sodium, mEq/L	136 ± 4	135 ± 5	0.076
Serum Potassium, mmol/L	4.4 (4.1–4.8)	4.2 (3.8–4.4)	0.384
Serum Calcium, mg/dL	9.2 (8.8–9.6)	8.5 (7.9–8.8)	0.011
Serum Albumin, g/L	3.6 (3.4–3.7)	3.2 (3.1–3.6)	0.036

**Table 4:** Comparison of expression levels of *NFKB1*, *SIRT1*, *NFE2L2*, and *FOXO1* in patients with/without contrast-induced nephropathy.

	All Patients N = 48	Group 2 N = 25	Group 1 N = 23	*p*-Value
*NFKB1*	0.78 (0.21–5.62)	0.24 (0.11–0.41)	5.7 (3.08–8.54)	<0.001
*SIRT1*	0.89 (0.56–1.78)	1.76 (0.66–3.24)	0.76 (0.5–0.92)	0.002
*NFE2L2*	0.79 (0.19–6.64)	0.2 (0.1–0.32)	6.8 (2.88–10.16)	<0.001
*FOXO1*	0.81 (0.18–4.94)	0.18 (0.1–0.24)	4.97 (3.04–8.84)	<0.001

**Table 5:** Univariate analysis of *NFKB1*, *SIRT1*, *NFE2L2*, and *FOXO1* expression levels to predict the risk of contrast-induced nephropathy progression.

	Univariate Analysis	
Variable	Odds Ratio (95% CI)	*p*-Value
*NFE2L2* (>1.34-fold)	26.32 (3.731–166.667)	<0.001
*NFKB1* (>0.78-fold)	11.5 (3.043–43.461)	<0.001
*FOXO1* (>1.32-fold)	(NA-NA)	<0.001
*SIRT1* (<0.88-fold)	2.36 (1.216–4.587)	0.004

## Data Availability

The raw data supporting the conclusions of this article will be made available by the authors on request.

## Abbreviations

The following abbreviations are used in this manuscript:

AKI	Acute kidney injury
BMI	Body Mass Index
CIN	Contrast-induced nephropathy
eGFR	Estimated Glomerular Filtration Rate
FOXO	Forkhead box gene
HO-1	Heme oxygenase-1
NF-Κb	Nuclear factor kappa B
NRF2	Nuclear factor erythroid-2-related factor 2
p-PCI	Primary percutaneous coronary intervention
ROC	Receiver Operating Characteristic
ROS	Reactive oxygen species
RT-PCR	Reverse Transcriptase-Polymerase Chain Reaction
